# Functional Studies on Novel *RET* Mutations and Their Implications for Genetic Counseling for Hirschsprung Disease

**DOI:** 10.3389/fgene.2019.00924

**Published:** 2019-10-08

**Authors:** Hui Wang, Qi Li, Zhen Zhang, Ping Xiao, Long Li, Qian Jiang

**Affiliations:** ^1^Department of Medical Genetics, Beijing Municipal Key Laboratory of Child Development and Nutriomics, Capital Institute of Pediatrics, Beijing, China; ^2^Department of General Surgery, Capital Institute of Pediatrics Affiliated Children’s Hospital, Beijing, China; ^3^Department of Pathology, Capital Institute of Pediatrics Affiliated Children’s Hospital, Beijing, China

**Keywords:** Hirschsprung disease, functional analysis, *RET*, splice site mutation, premature termination codon

## Abstract

Hirschsprung disease (HSCR) is a genetic disorder characterized by the absence of ganglion cells in the gut. *RET* is considered to be the main susceptibility gene. In our previous screening of 83 HSCR patients, targeted exome sequencing identified nine rare variants of *RET*, most of which were new discoveries. Here, we performed *in vitro* arrays with functional studies to investigate their effects. Two variants (p.R77C and p.R67insL) were demonstrated to disrupt the glycosylation of RET and affect its subcellular localization. Three nonsense mutations (p.W85X, p.E252X, and p.Y263X) could not produce detectable RET full-length protein, and the other three mutations (p.R770X, p.Q860X, and p.V778Afs*1) were translated into truncated proteins of predicted sizes. One canonical splice acceptor site mutation (c.2802-2 A > G) was verified to affect gene regulation through aberrant splicing. In addition, we explored the effects of read-through reagents on *RET* nonsense mutations and showed that G418 significantly increased the full-length RET protein expression of p.Y263X in a dose-dependent manner, together with a mild recovery of p-ERK and p-STAT3. Our data provide a functional analysis of novel *RET* mutations and suggest that all of the rare variants detected from patients with clinically severe HSCR are indeed pathogenic. Thus, our findings have implications for proper genetic counseling.

## Introduction

Hirschsprung disease (HSCR, MIM 142623), or congenital megacolon, was first described in 1888 by Dr. Harald Hirschsprung (1830–1916), a Danish pediatrician (Sergi, 2015). The average incidence of HSCR is 1 in 5,000 live births with a sex ratio of approximately four males/one female. However, the incidence varies significantly between different ethnic groups, and the highest incidence, namely, 1 in 3,571 live births, has been reported in Asians (Kenny et al., 2010). HSCR is defined as the absence of ganglion cells in the myenteric and submucosal plexuses of the intestinal wall with concomitant hypertrophy of parasympathetic nerve fibers (Sergi, 2015; Schriemer et al., 2016). The disorder is categorized into three types: short-segment (S-HSCR, aganglionosis segment up to the upper sigmoid colon, 80%), long-segment (L-HSCR, aganglionosis beyond the splenic flexure, 15%), and total colonic aganglionosis (TCA, 5%) (Chatterjee et al., 2016). Genetic studies demonstrate complex patterns of inheritance and variable expression, the causes of which remain largely undetermined (Borrego et al., 2013).

HSCR is associated with the abnormal migration, proliferation, and differentiation of enteric neural crest cells in the intestinal tract during the early development of the enteric nervous system (ENS). The ENS is the intrinsic nervous system of the bowel and regulates most aspects of bowel function (Hao et al., 2016). This process is regularly controlled by strongly related molecules, and genetic studies have identified rare, high-penetrance coding variants of 14 genes that together explain ∼10% of cases (Chatterjee et al., 2016). Among these, *RET* is the only gene to be considered a major player in HSCR because its mutations account for more than 80% of known pathogenic mutations, mainly consisting of loss-of-function (LOF) mutations and *de novo* mutations, detected in HSCR patients (Gui et al., 2017). The frequency of *RET* gene mutations in familial cases can be as high as 50%, and the ratio in sporadic HSCR patients is usually between 15% and 35%. Data from both targeted exome sequencing and whole-exome sequencing analyses suggest that deleterious *RET* variants are significantly enriched in HSCR patients compared with the normal population, reaffirming the vital role of RET in the disease (Jiang et al., 2017b).

*RET* proto-oncogene mutations are associated with many human diseases and are spread throughout the entire coding sequence (CDS). Gain-of-function gene mutations are closely related to the occurrence of multiple endocrine neoplasias (MENs), including MEN types 2A and 2B and familial medullary thyroid carcinoma (Boikos and Stratakis, 2008). In contrast, *RET* mutations found in HSCR patients are all LOF mutations (Carlomagno et al., 1996) and can be roughly divided into two groups: 1) null variants with the production of a truncated protein due to a nonsense mutation, frameshift insertion/deletion or canonical ±1 or 2 splice site mutation; and 2) variant of uncertain significance, such as a missense mutation, in-frame insertion/deletion, or noncanonical splice site mutation. To date, most of the *RET* mutations found in HSCR patients have been reported based only on *in silico* predictions, and a minority of the *RET* mutations has been identified in the context of functional consequences via *in vitro* assays. Furthermore, ∼35% of the *RET* variants that were functionally tested were ultimately demonstrated to be noncausative variants (Widowati et al., 2016). Notably, *RET*-CDS mutations are not fully penetrant, and assessments of their functional significance are truly essential, not only for understanding the mechanism of the disease but also for providing accurate genetic counseling and recurrence risk evaluations (Leon et al., 2012). Herein, we performed an unbiased study of nine HSCR-associated *RET* mutations (p.R77C, p.W85X, p.E252X, p.Y263X, p.R770X, p.Q860X, p.V778Afs*1, p.R67insL, and c.2802-2A > G) (Jiang et al., 2017b). *In vitro* cellular experiments confirmed LOF effects for all of them, and these effects were manifested as either disruption of RET phosphorylation or the production of a truncated protein with consequent subcellular mislocalization or aberrant splicing.

## Materials and Methods

### *In Silico* Prediction

To predict the functional impacts of the mutations, *in silico* analysis was performed using four online software programs: SIFT (http://sift.jcvi.org/), PROVEAN (http://provean.jcvi.org/protein_batch_submit.php?species=human), Polyphen 2 (http://genetics.bwh.harvard.edu/pph2/), and MutationTaster2 (http://www.mutationtaster.org/). The only intronic mutation, namely, c.2802-2A > G, was predicted by Human Splicing Finder 3.1 (http://www.umd.be/HSF3/HSF.shtml) and BDGP (http://www.fruitfly.org/seq_tools/splice.html).

### Plasmids

The human RET (RC202552) and GFRA1 (RG219943) expression vectors were purchased from OriGene Technologies (Rockville, MD, USA). All *RET* mutations were introduced into the wild-type isoform using a site-directed mutagenesis kit (QuikChange Lightning Site-Directed Mutagenesis Kit from Agilent, Santa Clara, CA, USA 210518). The whole sequence (wild-type (WT) or mutant) was confirmed by Sanger sequencing. The PCMV6-entry-GFP vector (constructed based on the GFRA1 vector by removing the *GFRA1* sequence) was used as a marker of transfected cells. The primers used in this assay are shown in **Table S1**.

### Minigene Assay

To determine the effects of a possible splice site variant (c.2802-2A > G), we introduced the wild-type and mutant intron 16 of *RET* into the wild-type sequence upstream of exon 17 and named the products RET-AG and RET-GG, respectively. HEK293 cells were grown in six-well plates and transfected with RET wild-type, RET constructs with normal (RET-AG) or abnormal (RET-GG) intron 16, or empty vector. RNA was extracted 24 h after transfection with TRIzol Reagent (Thermo Fischer Scientific, Waltham, MA, USA 15596026). EasyScript One Step gDNA Removal and cDNA Synthesis SuperMix (TransGen Biotech, Beijing, China) was used to reverse RNA to cDNA. PCR amplification of the targeted region (from exon 15 to exon 19) was carried out with TransTaq DNA Polymerase High Fidelity (AP131; TransGen Biotech, Beijing, China) with the following primers: c2802-2A > G cDNA F (5′-ggcaattgaatccctttttg-3′) and c2802-2A > G cDNA R (5′-cttccagcattgcagcatc-3′). Agarose gel electrophoresis and nucleotide sequencing of the PCR products were conducted to evaluate the effects of the splice site variant.

### Transfection, Western Blot Analysis, and Immunofluorescence Staining

After resuscitation and *in vitro* culture for two to three passages (within 10 generations), human embryonic kidney (HEK293T) cells (purchased from the National Infrastructure of Cell Line Resource, China) were plated at a density of 1 × 10^6^ into six-well plates which were coated with 0.1 mg/ml poly-d-lysine (Merck Millipore, Darmstadt, Germany) for 6 h at 37°C and washed three times with PBS. The cells were cultured in DMEM (Invitrogen, Carlsbad, CA, USA) with 10% fetal bovine serum (Gibco, 10099141), 1% penicillin/streptomycin (Gibco, 15140122), and 1% glutamine (Gibco, 25030081). All of the cells were cultured for 12 h at 37°C with 5% CO2 before transfection with 1.5-μg RET-WT or mutant, 0.6-μg GFRA1, and 0.4-μg GFP construct in 7.5 μl of Lipofectamine 2000 Transfection Reagent (Thermo Fischer Scientific, 11668019). Two previously reported RET variants (p.L56M and p.Y1062F) were used as negative (nonpathogenic) and positive (pathogenic) controls in the missense mutation functional experiments (Geneste et al., 1999). Twelve hours after transfection, we changed the culture medium to OPTI-MEM (Gibco, 1929947) for 36 h, and then, the cells were lysed by means of the One Step Animal Cell Active Protein Extraction Kit (Sangon Biotech, Shanghai, China), and the protein concentration was determined by the Coomassie Brilliant Blue method. The lysates were subjected to electrophoresis and transferred to nitrocellulose membranes (Merck Millipore, Darmstadt, Germany); for electrophoresis, 25 µg of total protein was separated on 10% SDS-polyacrylamide gels prior to electrotransfer. The membranes were then detected with primary antibodies: anti-RET, anti-STAT3, anti-ERK, anti-phosphorylated-RET (p-RET), anti-phosphorylated-STAT3 (p-STAT3), anti-phosphorylated-ERK (p-ERK), anti-GFP, anti-GDNF, and anti-GAPDH (the complete list of antibodies is provided in **Table S2**). A Western blotting analysis with the anti-RET N-terminal antibody (extracellular domain; epitopes corresponding to amino acids 31-330) revealed two bands corresponding to the fully glycosylated (mature protein, roughly 175 kDa) and nonglycosylated (immature protein, roughly 150 kDa) forms. Finally, we developed the blot using the ECL Prime Western Blotting Reagent (GE Healthcare, Buckinghamshire, UK). Western blot images were captured and analyzed using a Tanon 5200 chemiluminescent imaging system (Tanon, Shanghai, China). The densities of the RET, p-RET, and p-ERK bands for all conditions were normalized to the densities of the corresponding GFP and GAPDH signals. The normalized p-RET, p-STAT3, and p-ERK densities were further normalized to the normalized RET values. Three independent experiments were performed for each condition.

For immunofluorescence staining, 100 HEK293 cells were grown on coverslips, which had been sterilized and poly--lysine-coated for 6 h, and transiently transfected with 0.4 μg of RET plasmid using 2 μl of lipofectamine 2000 transfection reagent according to the manufacturer’s instructions. Forty-eight hours later, cells were fixed in 4% paraformaldehyde for 10 min at room temperature and rinsed three times with 1 × PBS, each for 5 min. Cells were blocked with blocking buffer (5% bovine serum albumin in 1 × PBS) for 1 h and incubated overnight at 4°C with anti-RET antibody (the list of antibodies is provided in **Table S2**). Cells were washed three times with PBST (5 min/each) and incubated for 1 h at room temperature with Alexa Fluor^®^ 488-conjugated goat anti-mouse IgG (H+L) (ZSGB-BIO, Beijing, China). Cells were washed and incubated for 30 min with 100 nm phalloidin (cytoskeleton, Denver, CO, USA) at room temperature and washed three times with PBST. After adding a small amount of DAPI for 10 min, the coverslips were placed on a slide and visualized on a confocal laser scanning microscope (Leica, Mannheim, Germany). Three independent experiments were performed for each condition.

### G418 Suppression Assay

HEK293T cells (subjected to the previously described culture conditions and passage number) were seeded at 800 cells *per* well in six-well plates. After 12 h, the transfected cells were starved by changing the medium to OPTI-MEM and then simultaneously treated with different concentrations of G418, namely, 0, 0.4, 0.8, 1.2, 1.6, or 2 mg/ml, or with gradient concentrations of PTC124, namely, 0, 2.5, 5, 7.5, 10, or 12.5 µg/ml for 48 h. Cells were finally lysed and analyzed by Western blot. Agents used in this study are provided in **Table S3**.

### Statistical Analysis

The densitometry results of the Western blot analysis are presented as the mean ± standard deviation. Differences in the densities of p-RET, p-STAT3, and p-ERK for the RET mutant condition compared with the RET-WT condition were examined using analysis of variance. Differences were determined to be significant when the *P* value is less than 0.05.

## Results

### Rare Variants of *RET* Investigated in the Current Study and the *in Silico* Prediction

In our previous screening of 83 HSCR patients, targeted exome sequencing identified nine rare variants of *RET* (Jiang et al., 2017b): six were LOF mutations [c.254G > A (p.Trp85X), c.754G > T (p.E252X), c.789C > G (p.Y263X), c.2308C > T (p.R770X), c.2333delT (p.V778Afs*1), and c.2578C > T (p.Q860X)], one was a canonical splice acceptor site mutation (c.2802-2A > G), one was a previously reported pathogenic missense mutation [c.229C > T (p.R77C)], and one was likely pathogenic due to an in-frame insertion [c.200insTCC (p.R67insL)]. The affected amino acids of five variants (p.R67insL, p.R77C, p.W85X, p.E252X, and p.Y263X) are located in the cadherin domain of the RET extracellular module, while the other four (p.R770X, p.Q860X, p.V778Afs*1, and c.2802-2A > G) are in the TK domain (Jiang et al., 2017b). Most of these variants have never been reported, and functional studies are nonexistent (**Table 1**).

**Table 1:** *RET* rare variants investigated in this study.

Nucleotide change	Amino-acid change	Exon/intron	Frequency			Sex Type	Inheritance	Position (hg19)
			1000G	gnomAD	Inhouse			
c.229C > T	p.R77C	exon 2	0	0	0	M/TCA	Paternal mosaicism	chr10: 43596062: C > T
c.254G > A	p.W85X	exon 2	0	0	0	M/TCA	Maternal mosaicism	chr10: 43596087: G > A
c.754G > T	p.E252X	exon 4	0	0	0	M/TCA	*de novo* (postzygotic)	chr10: 43600528: G > T
c.789C > G	p.Y263X	exon 4	0	0	0	F/L-HSCR	Paternal mosaicism	chr10:43600563: C > G
c.2308C > T	p.R770X	exon 13	0	0	0	M/TCA	*de novo*	chr10:43613844: C > T
c.2578C > T	p.Q860X	exon 14	0	0	0	M/TCA	Maternal mosaicism	chr10:43615164: C > T
c.2333delT	p.V778Afs*1	exon 13	0	0	0	M/S-HSCR	*de novo*	chr10: 43613869: delT
c.200insTCC	p.R67insL	exon 2	0	0	0	F/TCA	*de novo* (postzygotic)	chr10: 43596029: insTCC
c.2802-2A > G	–	intron 16	0	0	0	F/S-HSCR	*de novo*	chr10: 43619117: A > G

F, female; M, male; S-HSCR, short-segment Hirschsprung disease; L-HSCR, long-segment Hirschsprung disease; TCA, total colonic aganglionosis.

We first applied *in silico* analysis to predict the possible functional impacts of the only missense variant and the splice site mutation. p. Arg77Cys was predicted to be damaging by SIFT with a score of 0.004, as well as by PolyPhen-2 (probably damaging, score 0.866). With respect to the intronic variation, c.2802-2A > G, both Human Splicing Finder and the BDGP program predicted that it was highly likely to affect splicing by removing the original splicing site.

### Minigene Assay

**Figure 1:**
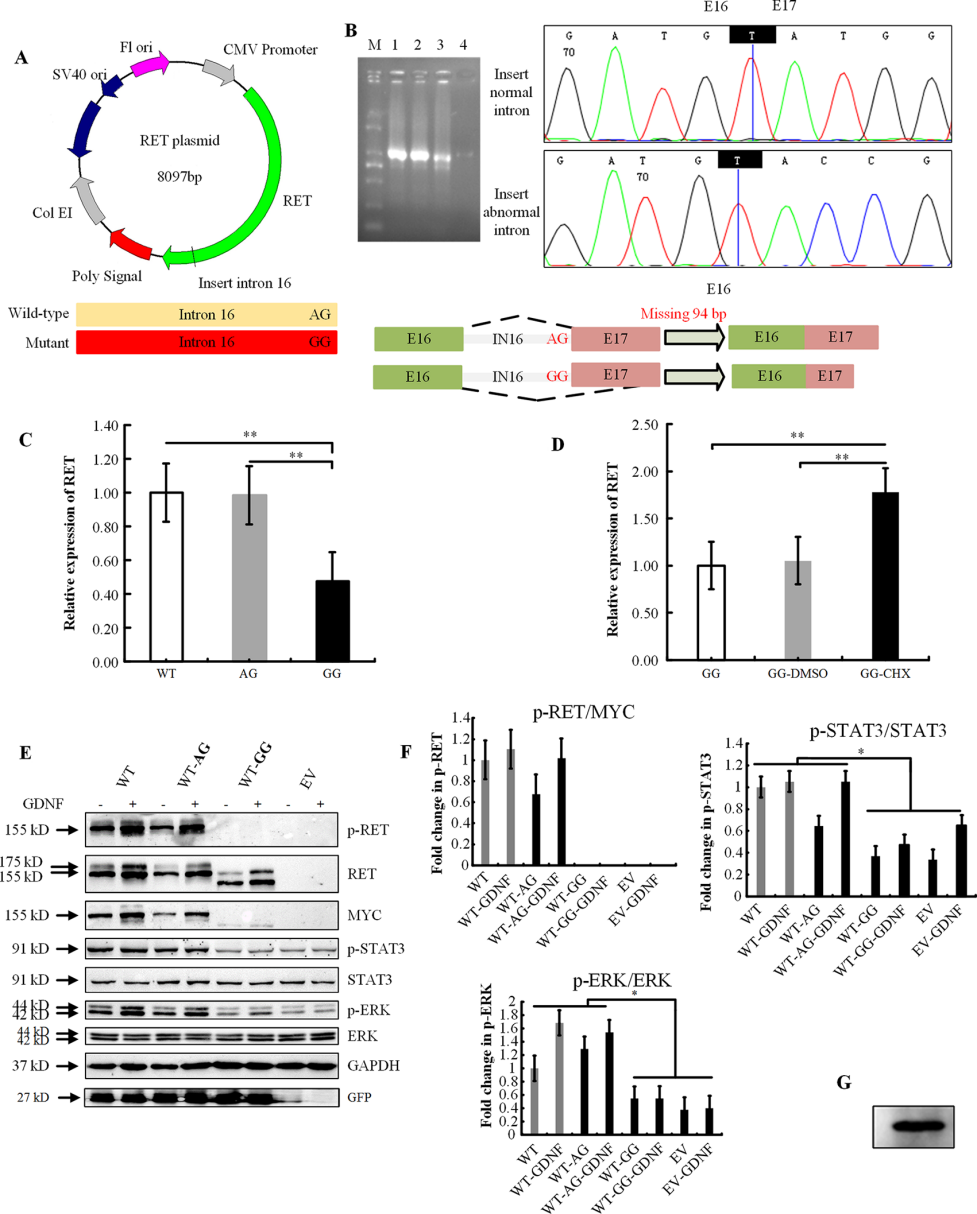
Functional analysis of the *RET* splicing mutation. **(A)** Schematic representation of the wild-type and mutant constructs used to examine the impact of splicing at exon 17. The important bases are marked. **(B)** Agarose gel analysis of the PCR amplification products after reverse transcription and sequence analysis of the bands obtained for the wild-type and the c.2802-2A > G mutant constructs. The latter shows a double band that consists of a major form that is missing 94 bp of exon 17. M: DNA marker (Tiangen Biotech); 1: RET-WT construct; 2: RET-AG construct; 3: RET-GG construct; 4: empty vector; E: exon; IN: intron. **(C)** Quantification of the *RET* RT-PCR products obtained from HEK293 cells after transfection. WT, RET wild-type plasmid as the control; AG, RET-wild-type plasmid with normal intron 16; GG, RET wild-type plasmid with abnormal intron 16 c.2802-2A > G. The results show the average of three independent experiments. Error bars indicate the standard deviation, and *P* values were calculated using Student’s independent *t*-test (**P < 0.001). **(D)** Quantification of the RET RT-PCR products obtained from HEK293 cells after transfection and treatment with cycloheximide (CHX, 100 µg/ml). CHX inhibited NMD (nonsense-mediated mRNA decay) and increased the mRNA level of RET (**P < 0.001). **(E)** Western blot analysis of whole cell lysates from HEK293T cells transiently transfected with RET-WT, RET-constructs RET-AG, RET-GG, or empty vector (EV). Protein from cells that were serum-starved 48 h prior to activation was resolved on 10% SDS-PAGE gels, and membranes were immunoblotted for the indicated proteins and phosphoproteins. (+), with GDNF treatment; and (-), without GDNF treatment. **(F)** Densitometric analysis of p-RET, MYC, p-STAT3, STAT3, p-ERK, and ERK. Values were normalized using GFP and GAPDH. Three independent experiments were performed for each condition (N = 3). (*P < 0.05). **(G)** Western blot analysis of whole cell lysates from HEK293T cells indicating endogenous expression of GDNF.

The intronic variation c.2802-2A > G was predicted to alter the normal splice acceptor site upstream of exon 17. To test this hypothesis, we constructed a RET plasmid consisting of exons 1 to 16 and intron 16, followed by exons 17 to 20 (**Figure 1**). HEK293 cells were transfected with RET-WT, RET-AG, RET-GG, or empty vector. Twenty-four hours after transfection, we harvested the cells, extracted the RNA, and reversed transcribed the RNA into cDNA. Agarose gel electrophoresis showed that both the RET-WT construct and RET construct with normal intron 16 produced a single band of equal size, as expected, while the mutant construct produced a smaller and dimmer band than the RET-WT construct (**Figure 1**). By sequencing the PCR products, we confirmed that both RET-WT and RET-AG produced a normal mRNA of *RET*, whereas RET-GG produced an abnormal form: a shorter transcript with 94 bp missing in exon 17. This sequence skipping was predicted to produce a frameshift mutation and finally introduce a premature termination codon (PTC), p.Val934Valfs*18. Given the appearance of the PTC, we hypothesized that this aberrant splicing may encode a C-terminal truncated RET protein. The q-PCR results showed that the mRNA expression level of the abnormal *RET* splicing variant was indeed significantly lower than those of the control constructs (RET-WT and RET-AG) (**Figure 1**). To further validate our hypothesis, we next transfected the RET construct with abnormal intron 16 (RET-GG) into HEK293 cells and treated the cells with cycloheximide (CHX) 12 h after transient transfection. As displayed in **Figure 1**, CHX effectively increased the mRNA level of the RET aberrant splicing variant (*P* < 0.05), indicating that the decreased expression level of *RET* mRNA is related to the trigger of nonsense-mediated mRNA decay. Finally, aberrant splicing was demonstrated to produce a truncated RET protein that severely disrupted the phosphorylation of STAT3 and ERK (**Figures 1E, F**). The activated phosphorylation form of RET observed without GDNF treatment (in **Figure 1**) prompted us to hypothesize that endogenous expression of GDNF occurs. Western blot analysis of the whole cell lysates from the HEK293T cells confirmed this hypothesis (**Figure 1**). Thus, GDNF activation was revoked in all subsequent experiments.

### Effect of the Mutation on Protein Glycosylation, Phosphorylation, and Localization

The wild-type and other eight mutant constructs were transiently transfected into HEK293T cells, which do not endogenously express the RET protein. Two bands were observed for the RET-WT with the anti-RET N-terminal antibody; however, only one band at 155 kDa was detected for RET-WT with the C-terminal MYC-tag antibody. We speculate that glycosylation of the RET protein may have modified the MYC amino acid sequence, so the encapsulated protein could not be detected by the antibody.

**Figure 2:**
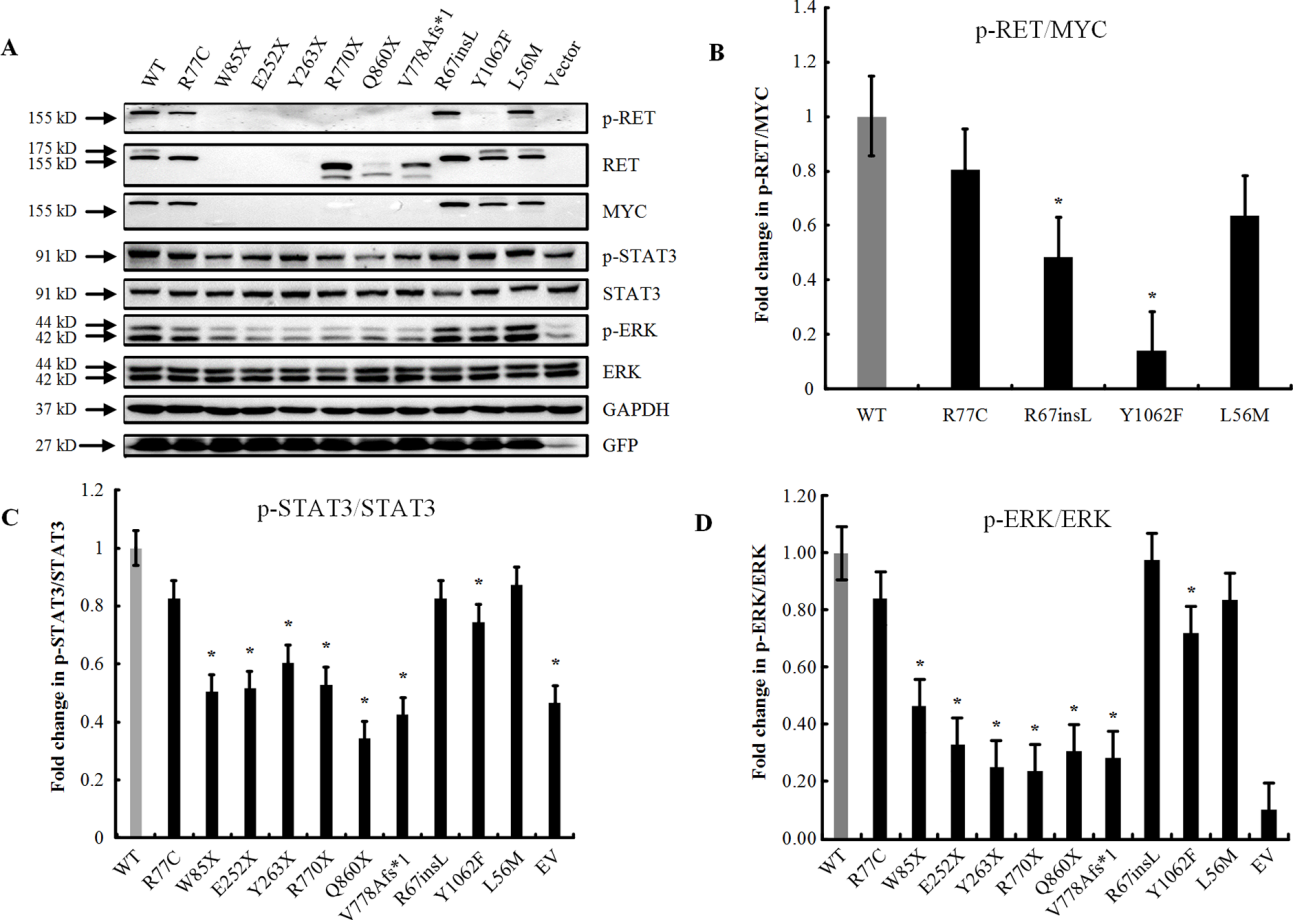
Effect of the mutation on RET glycosylation and phosphorylation. **(A)** Western blot analysis of the lysates of HEK293T cells transfected with RET-WT or RET-mutants: p.R77C, p.W85X, p.E252X, p.Y263X, p.R770X, p.Q860X, p.V778Afs*1, p.R67insL, and empty vector (EV). Two mutants were included in this experiment as positive (p.Y1062F) and negative (p.L56M) controls. **(B)** Densitometric analysis of p-RET and MYC. Values were normalized using GFP and GAPDH. **(C)** Densitometric analysis of p-STAT3 and STAT3. Values were normalized using GFP and GAPDH. The expression of p-STAT3 is shown as the fold change relative to STAT3 expression. **(D)** Densitometric analysis of p-ERK and ERK. Values were normalized using GFP and GAPDH. The expression of p-ERK is shown as the fold change relative to ERK expression. Three independent experiments were performed for each condition (N = 3, *P < 0.05).

The missense variant p.R77C and in-frame insertion p.R67insL produced a full-length RET protein, but only the nonglycosylated isoform at 155 kDa could be detected. No band was detected with either the N-RET or C-terminal MYC-tag antibodies in the p.W85X-, p.E252X-, or p. Y263X-transfected cells. For the other two nonsense mutations (p.R770X, p.Q860X) and the frameshift mutation p.V778Afs*1, no band was detected with the C-terminal MYC antibody, while two bands of similar size, both smaller than the RET-WT bands, were detected with the N-RET antibody (**Figure 2**), indicating that these three mutations produced a C-truncated RET protein. As expected, all mutations with PTC had significantly lower levels of p-STAT3 and p-ERK, and no p-RET bands were detectable. The in-frame insertion p.R67insL and the positive control p.Y1062F had significantly lower levels of p-RET than RET-WT. In contrast, the missense mutation p.R77C and the negative control p.L56M showed no significant differences in the phosphorylation levels of the RET, STAT3, and ERK proteins (**Figures 2B–D**).

**Figure 3:**
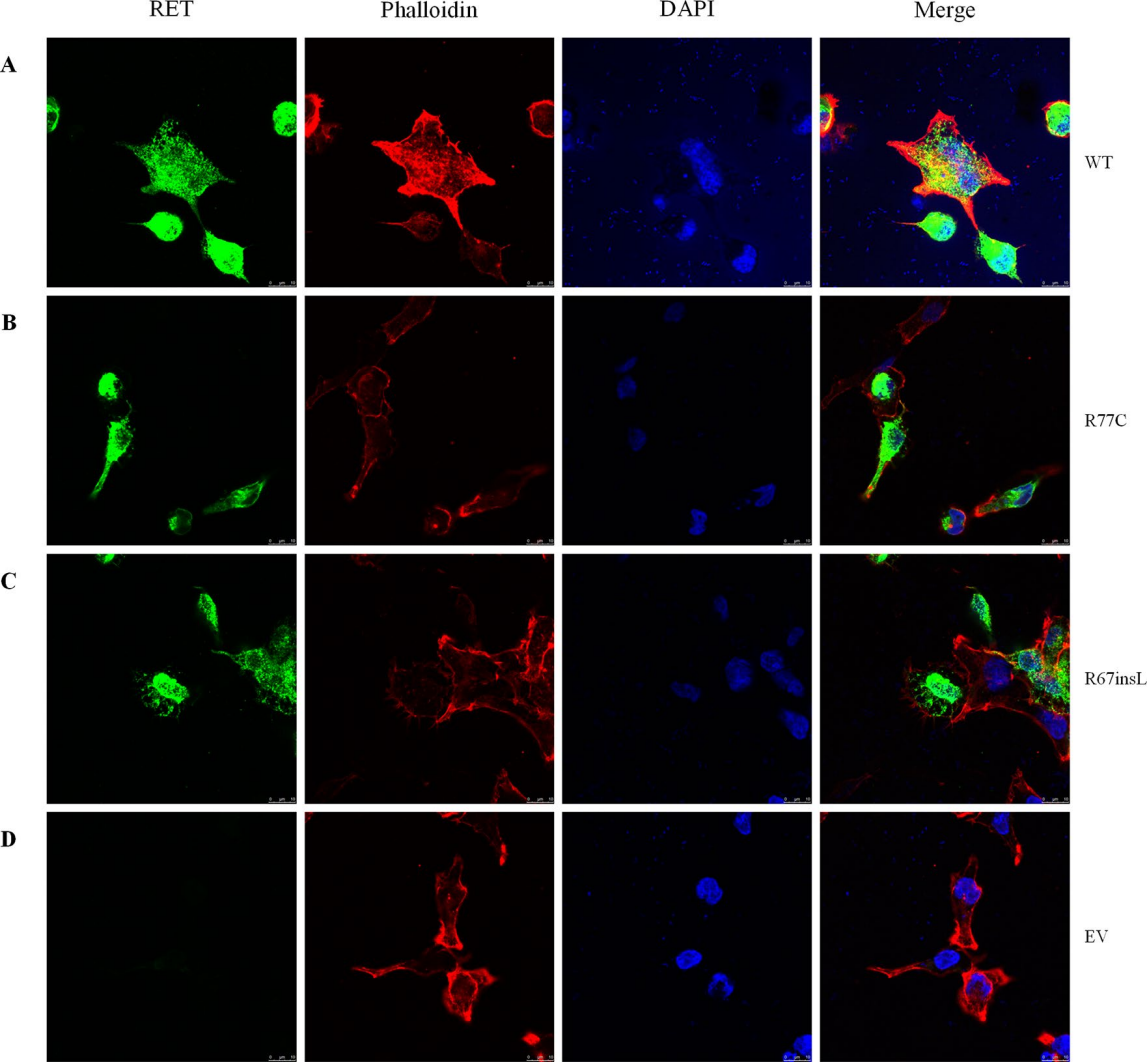
The localizations of RET mutant proteins. Immunofluorescence staining utilizing a primary antibody against RET upon transfection of HEK293 cells with **(A)** RET-WT, **(B)** RET-R77C, **(C)** RET-R67insL, or **(D)** empty vector (EV). RET-WT staining shows strong signals in both the cytoplasm and cell membrane, whereas the expression of RET-R77C and RET-R67insL is less pronounced on the cell membrane and is localized closer to the nucleolus/endoplasmic reticulum.

Furthermore, we performed immunofluorescence staining to analyze the subcellular localizations of the mutant proteins. Double-staining was performed with both C-RET and phalloidin antibody to visualize the cell shape and the relative position of the RET protein. Compared with RET-WT, the rare extracellular variants p.R77C and p.R67insL showed an absence of glycosylated (mature) RET protein (**Figure 3**).

### Dosage-Dependent Effects of the RET Mutations

**Figure 4:**
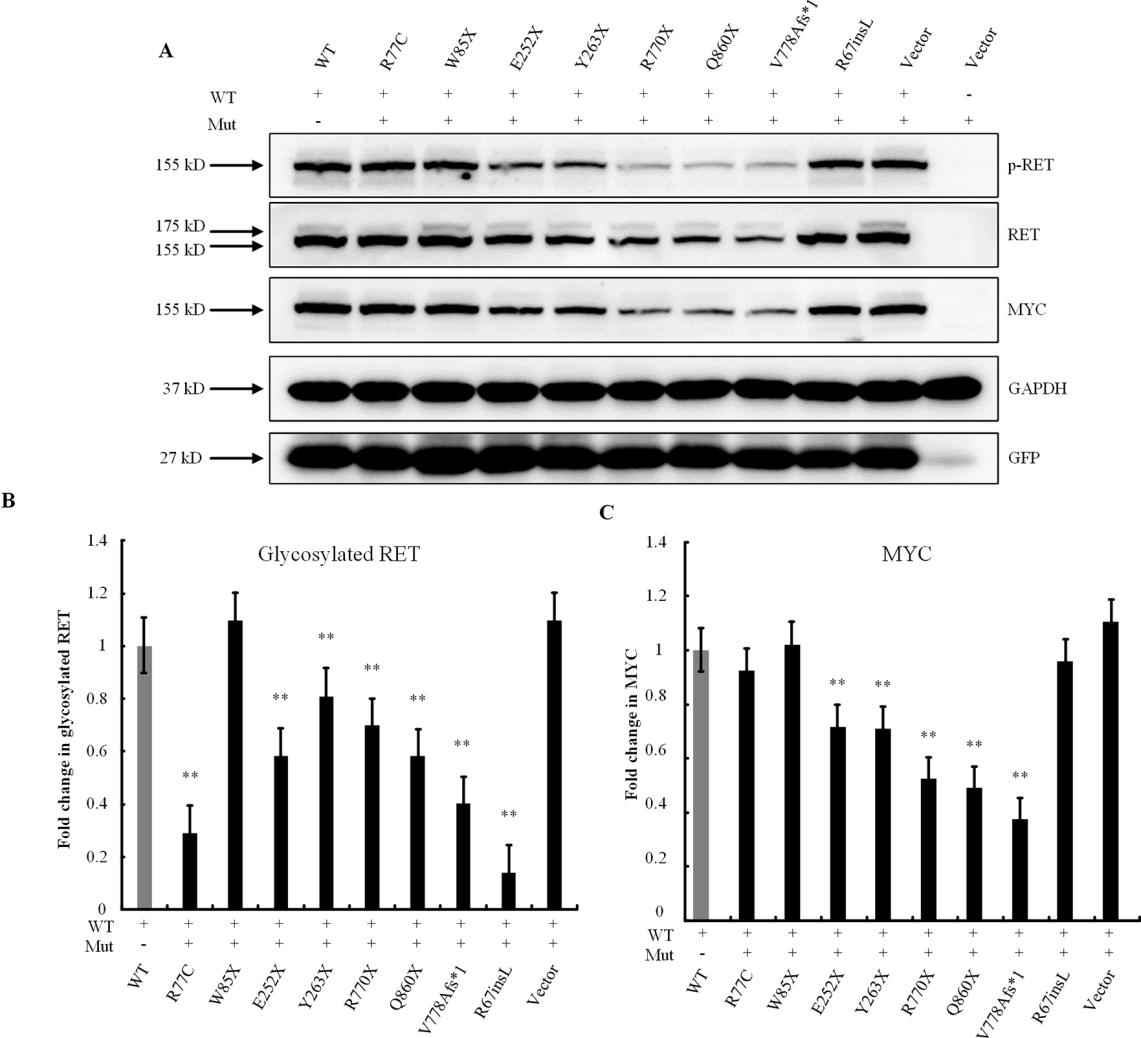
Dose-dependent effects of *RET* mutations. **(A)** Western blot analysis of the lysates of HEK293T cells transfected with RET-WT or cotransfected with RET-WT and a RET-mutant construct, namely, p.R77C, p.W85X, p.E252X, p.Y263X, p.R770X, p.Q860X, p.V778Afs*1, p.R67insL, or empty vector (EV). **(B)** Densitometric analysis of glycosylated RET protein. Values were normalized using GFP and GAPDH. **(C)** Densitometric analysis of MYC. Values were normalized using GFP and GAPDH (**P < 0.01).

The RET-WT and RET-Mut plasmids were then transfected simultaneously at a 1:1 ratio to examine dose-dependent effects. Briefly, cells were transiently transfected with 1.5 μg of RET plasmid (0.75 μg of RET-WT and 0.75 μg of RET-Mut), 0.6 μg of GFRA1, and 0.4 μg of GFP construct in 7.5 μl of Lipofectamine 2000 Transfection Reagent. The cell lysates were subjected to Western blotting analysis. Both the missense variant p.R77C and the in-frame insertion p.R67insL showed significantly lower levels of glycosylated RET protein (**Figure 4**). The expressions of RET protein in the p.R770X-, p.Q860X-, and p.V778Afs*1-transfected cells, which contain PTCs and may produce C-truncated RET proteins, were significantly lower than that in the RET-WT-transfected cells (**Figure 4**), indicating that these three mutants affected the production of normal RET protein. Surprisingly, the expression level of the RET nonsense mutation, namely, p.W85X was not significantly different from that of RET-WT. Also, the other nonsense mutations, namely, p.E252X and p.Y263X, were slightly reduced from that of RET-WT (**Figures 4A, C**).

### Premature Termination Codon Rescue With G418

**Figure 5:**
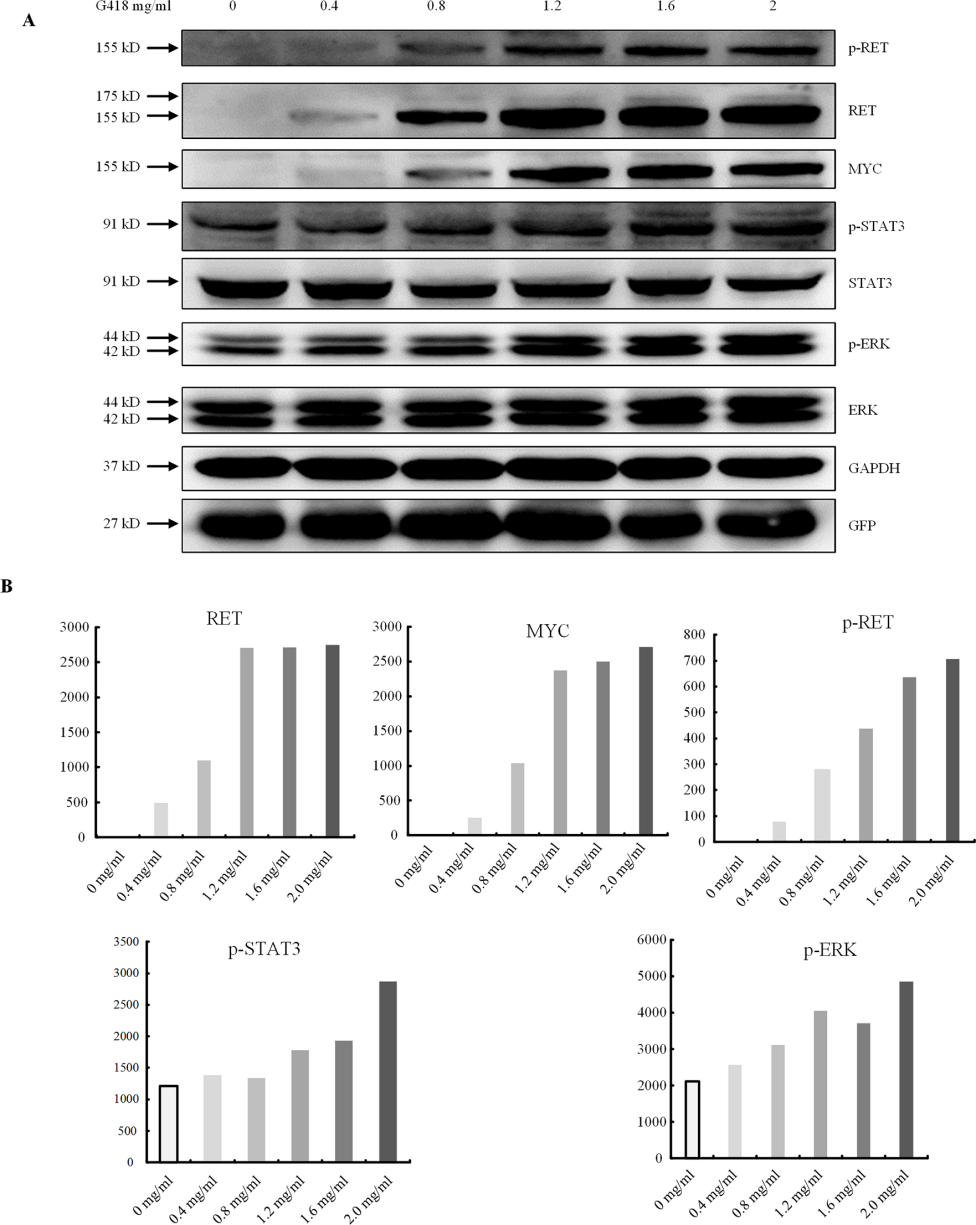
Premature termination codon rescue with G418. **(A)** The dose-dependent effect of read-through rescue of p.Y263X with G418 was assessed using western blotting. Values were normalized using GFP and GAPDH. GAPDH was used as the internal control. **(B)** Cells were transfected with plasmids carrying the p.Y263X nonsense mutant and then treated with different doses of G418.

Finally, to determine whether PTC-containing mutations could be rescued by read-through reagents, we transfected the indicated mutant RET plasmid with GFRA1 and GFP plasmids into HEK293T cells, replaced the original culture medium with OPTI-MEM 12 h after transfection, and subjected these cells to a gradient treatment of G418 or PTC124. Western blotting analysis showed that G418 had a significant rescue effect on p.Y263X, whereby all bands for p-RET, p-STAT3, and p-ERK were detectable (**Figure 5**). In addition, a certain degree of RET activity recovery was also observed (**Figure 5**). In contrast, the expression levels of the RET full-length proteins for p.R770X, p.Q860X, and p. V778Afs* 1 were not increased by G418, even as the G418 concentration increased (data not shown). In addition, the RET expression levels for p.W85X and p.E252X showed only marginal recovery, as did p-RET, p-ERK, and p-STAT3 levels (data not shown). Finally, after treatment with PTC124, these six mutations showed no significant increases in the expression levels of RET full-length protein (data not shown).

## Discussion

In this study, we report nine rare *RET* mutations found in 87 HSCR patients, including one missense mutation, one splice site mutation, one in-frame insertion, one frameshift mutation, and five nonsense mutations (Jiang et al., 2017b). Functional studies demonstrated that these identified mutations all disrupted RET biological functions via various mechanisms: 1) two extracellular domain mutations, namely, p.R77C and p.R67insL, disrupted protein glycosylation, and the lack of glycosylation of the RET protein resulted in cellular mislocalization (Kjaer and Ibanez, 2003); and 2) five nonsense mutations, one frameshift mutation, and a splice site mutation, all of which produced truncated proteins, had abnormal RET function and activity. Interestingly, the activity of the nonsense mutation p.Y263X could be rescued by the read-through reagent G418.

Missense mutations of the extracellular domain of RET can decrease the level of RET glycosylation (Carlomagno et al., 1996), interfering with its maturation, hindering its transport to the cell membrane and disrupting the binding site of RET for subsequent ligand binding (Widowati et al., 2016; Plaza-Menacho et al., 2006). Although the p-STAT3 and p-ERK levels of p.R77C and p.R67insL were not significantly decreased compared with those of RET-WT, the mutations both led to nonglycosylation of RET and, consequently, their mislocalization due to incorrect folding (Kjaer and Ibanez, 2003). We confirmed this by immunofluorescence staining, which showed that these two mutant proteins were localized close to the nucleus and the endoplasmic reticulum compared with the RET-WT protein. In support of our results, the extracellular variant p.P270L was previously demonstrated to disrupt the maturation of the RET protein with nonsignificantly lower p-ERK levels (Widowati et al., 2016). Interestingly, a strong reduction of glycosylated RET for p.R77C and p.R67insL was observed in the case of the 1:1 RET wild type and mutant plasmid transfection, even when compared with the results of the truncating variants (**Figure 4**). This phenomenon might related to the mediation of normal RET protein glycosylation by glycosylphosphatidylinositol (GPI) (Traugott and Moley, 2010) based on the following steps: a GPI anchor precursor is generated at the ER membrane, attached in the ER lumen by a GPI-transamidase complex to newly synthesized RET protein and then modified by several remodeling enzymes. This remodeling process will convert the GPI anchor into a transport signal that actively promotes the ER export of GPI-anchored protein to the Golgi apparatus, where they are subsequently routed to their functional site of residence, i.e., the plasma membrane (Muniz and Riezman, 2016). The mutant RET proteins p.R77C and p.R67insL may dominantly occupy the GPI complex, interfere with the biological binding/modification process, and thus lead to the decreased efficiency of RET protein glycosylation. Nevertheless, the exact mechanisms underlying the effects of these two mutations on RET function require further investigation.

Four of the seven mutations, namely, p.R770X, p.Q860X, p.V778Afs*1, and c.2802-2A > G, which contain PTC mutations, could produce a C-truncated protein and could be detected by the N-RET antibody at their corresponding sizes. These four truncated mutations severely affected the activity of RET and the downstream proteins p-ERK and p-STAT3. RET splice site mutations (Auricchio et al., 1999) have been reported in children with HSCR, but many splice site mutations, such as c.1880-4 A > G, are located outside the classical splice site (Widowati et al., 2016). In addition, some exon mutations, such as c.1947G > A (p. S649S) and c.1941C > T (p. I647I), may also affect the splice site (Auricchio et al., 1999). However, few studies have been performed to determine the real splicing consequences of these variants of *RET* (Widowati et al., 2016; Auricchio et al., 1999; Bolk et al., 2000). In this case, the splice site mutation in intron 16, namely, c.2802-2A > G, abolished the canonical “AG” acceptor site, which is necessary for correct splicing. The HSF3.1 software predicts that the mutation site disrupts the donor site during the original splicing process. *In vitro* experiments showed that the original splice site was destroyed by the mutation, resulting in a skipping of 94 bases of mRNA. In addition, abnormal splicing also introduces a PTC, which generates a truncated RET protein. Therefore, splice site abnormalities can also affect RET protein function and lead to HSCR, which is consistent with previous studies (Borrego et al., 2013; Basel-Vanagaite et al., 2007).

An important finding of this study is the positive response of the nonsense mutation p.Y263X to the read-through reagent G418. Aminoglycoside antibiotics are known to be able to induce the read-through of PTCs in cases caused by gene mutations (Prokhorova et al., 2017; Pranke et al., 2018). Common reading reagents include gentamicin, G418, and PTC124, among others. Gentamicin has the side effects of nephrotoxicity (McWilliam et al., 2017) and ototoxicity (Jiang et al., 2017a), and G418 is the most active and effective read-through drug. In 2014, the European Medicines Agency granted PTC124 a conditional marketing authorization (Shimizu-Motohashi et al., 2019). Here, we used G418 and PTC124 to treat *RET* containing PTC mutations for the first time, trying to rescue the function of the mutant RET. As a result, the premature termination produced by p.Y263X were overcome with detectable expression of the full-length protein, including both glycosylated and nonglycosylated RET, after G418 treatment, and p-RET and p-ERK were also recovered. These results demonstrated that RET protein function was partially restored after G418 treatment. Another notable point is that the other RET mutations with PTCs showed no obvious response to G418. These results suggested that the mutation site was a significant factor in determining the rescue efficiency according to a previous study (Yu et al., 2014). Although the read-through effect of PTC124 was demonstrated in a clinical trial in CF patients carrying nonsense mutations (Kerem et al., 2008). The results of our *in vitro* studies demonstrated read-through induction only with aminoglycosides and with not PTC124 (Ho et al., 2013; Brumm et al., 2012; Dranchak et al., 2011), suggesting the possible specificity of PTC124 for particular genes.

In this study, we tested only the glycosylation and phosphorylation of RET protein and examined downstream signaling of RET via the ERK and STAT3 proteins. Notably, RET also activated many other downstream targets that were not included in this study. In addition, HSCR is a complex disease, and functional studies of HSCR are challenging. In most cases, the disease is caused by a variety of variants, all likely to have effects. All of the nine rare *RET* mutations from the 83 HSCR patients examined in this study were confirmed to be pathogenic, whereas in previous studies, 36 (61%) of 59 RET variants (Widowati et al., 2016; Sribudiani et al., 2018) were considered to be disease-causing variants. The frequency of pathogenic mutations varies widely, perhaps because all of our variants were from severe cases (L-HSCR and TCA). There is no doubt that functional verifications of *RET* gene mutations are necessary for the genetic counseling of parents with HSCR. We may rely solely on website predictions to roughly assess the pathogenicity of RET mutations, but the final determination depends on validation by a RET functional study. Without functional verification, we should be cautious about the genetic counseling of parents. It is recommended that when new *RET* mutations are found, functional studies should be carried out in time for clinical decision making, such as prenatal diagnosis.

In conclusion, we performed *in vitro* functional studies of nine rare *RET* mutations, most of which were newly discovered. Two mutations produced full-length RET protein but only in the nonglycosylation form, which led to abnormal cellular localization. One splice site mutation caused aberrant splicing with the introduction of a new PTC. This mutation ultimately produced a truncated protein, as did the other three mutations (p.R770X, p.Q860X, and p.V778Afs*1), which showed no RET activity. The remaining three nonsense mutations (p.W85X, p.E252X, and p.Y263X) could not produce a detectable RET protein. Interestingly, the read-through drug G418 restored the function of the mutant protein by suppressing PTCs in transiently expressed cDNAs that contained the p.Y263X mutation in the *RET* gene, indicating that patient with HSCR caused by certain nonsense *RET* mutations may be considered for read-through drug therapy.

## Data Availability Statement

The raw data supporting the conclusions of this manuscript will be made available by the authors, without undue reservation, to any qualified researcher.

## Author Contributions

QJ conceived the project and planned the experiments. HW, QL, ZZ, PX, and LL clinically characterized the cases and collected blood samples. HW performed all the *in vitro* experiments. HW and QJ collected and assembled the data. All authors contributed to writing the manuscript and approved the final version.

## Funding

This study was supported by the National Natural Science Foundation of China (81300266, 81771620, and 81700451), the Beijing Natural Science Foundation (7142029), and the Beijing Nova Program (Z171100001117125).

## Conflict of Interest

The authors declare that the research was conducted in the absence of any commercial or financial relationships that could be construed as a potential conflict of interest.

## References

[B1] Auricchio A. Griseri P. Carpentieri M. L. Betsos N. Staiano A. Tozzi A. (1999). Double heterozygosity for a RET substitution interfering with splicing and an EDNRB missense mutation in Hirschsprung disease. Am. J. Hum. Genet. 64 (4), 1216–1221. 10.1086/302329 10090908 PMC1377847

[B2] Basel-Vanagaite L. Pelet A. Steiner Z. Munnich A. Rozenbach Y. Shohat M. (2007). Allele dosage-dependent penetrance of RET proto-oncogene in an Israeli-Arab inbred family segregating Hirschsprung disease. Eur. J. Hum. Genet. 15 (2), 242–245. 10.1038/sj.ejhg.5201733 17091122

[B3] Boikos S. A. Stratakis C. A. (2008). Molecular mechanisms of medullary thyroid carcinoma: current approaches in diagnosis and treatment. Histol. Histopathol. 23 (1), 109–116. 10.14670/hh-23.109 17952863

[B4] Bolk S. Pelet A. Hofstra R. M. Angrist M. Salomon R. Croaker D. (2000). A human model for multigenic inheritance: phenotypic expression in Hirschsprung disease requires both the RET gene and a new 9q31 locus. Proc. Natl. Acad. Sci. U. S. A. 97 (1), 268–273. 10.1073/pnas.97.1.268 10618407 PMC26652

[B5] Borrego S. Ruiz-Ferrer M. Fernandez R. M. Antinolo G. (2013). Hirschsprung's disease as a model of complex genetic etiology. Histol. Histopathol. 28 (9), 1117–1136. 10.14670/HH-28.1117 23605783

[B6] Brumm H. Muhlhaus J. Bolze F. Scherag S. Hinney A. Hebebrand J. (2012). Rescue of melanocortin 4 receptor (MC4R) nonsense mutations by aminoglycoside-mediated read-through. Obesity (Silver Spring, Md) 20 (5), 1074–1081. 10.1038/oby.2011.202 21738238

[B7] Carlomagno F. De Vita G. Berlingieri M. T. de Franciscis V. Melillo R. M. Colantuoni V. (1996). Molecular heterogeneity of RET loss of function in Hirschsprung's disease. EMBO J. 15 (11), 2717–2725. 10.1002/j.1460-2075.1996.tb00632.x 8654369 PMC450207

[B8] Chatterjee S. Kapoor A. Akiyama J. A. Auer D. R. Lee D. Gabriel S. (2016). Enhancer variants synergistically drive dysfunction of a gene regulatory network In Hirschsprung disease. Cell 167 (2), 355–368. 10.1016/j.cell.2016.09.005 27693352 PMC5113733

[B9] Dranchak P. K. Di Pietro E. Snowden A. Oesch N. Braverman N. E. Steinberg S. J. (2011). Nonsense suppressor therapies rescue peroxisome lipid metabolism and assembly in cells from patients with specific PEX gene mutations. J. Cell. Biochem. 112 (5), 1250–1258. 10.1002/jcb.22979 21465523 PMC3136445

[B10] Geneste O. Bidaud C. De Vita G. Hofstra R. M. Tartare-Deckert S. Buys C. H. (1999). Two distinct mutations of the RET receptor causing Hirschsprung's disease impair the binding of signalling effectors to a multifunctional docking site. Hum. Mol. Genet. 8 (11), 1989–1999. 10.1093/hmg/8.11.1989 10484767

[B11] Gui H. Schriemer D. Cheng W. W. Chauhan R. K. Antinolo G. Berrios C. (2017). Whole exome sequencing coupled with unbiased functional analysis reveals new Hirschsprung disease genes. Genome Biol. 18 (1), 48. 10.1186/s13059-017-1174-6 28274275 PMC5343413

[B12] Hao M. M. Foong J. P. Bornstein J. C. Li Z. L. Vanden Berghe P. Boesmans W. (2016). Enteric nervous system assembly: functional integration within the developing gut. Dev. Biol. 417 (2), 168–181. 10.1016/j.ydbio.2016.05.030 27235816

[B13] Ho G. Reichardt J. Christodoulou J. (2013). In vitro read-through of phenylalanine hydroxylase (PAH) nonsense mutations using aminoglycosides: a potential therapy for phenylketonuria. J. Inherited Metabol. Dis. 36 (6), 955–959. 10.1007/s10545-013-9602-6 23532445

[B14] Jiang M. Karasawa T. Steyger P. S. (2017a). Aminoglycoside-induced cochleotoxicity: a review. Front. Cell. Neurosci. 11, 308. 10.3389/fncel.2017.00308 29062271 PMC5640705

[B15] Jiang Q. Liu F. Miao C. Li Q. Zhang Z. Xiao P. (2017b). RET somatic mutations are underrecognized in Hirschsprung disease. Genet. Med. 20 (7), 770–777. 10.1038/gim.2017.178 29261189 PMC7814876

[B16] Kenny S. E. Tam P. K. Garcia-Barcelo M. (2010). Hirschsprung's disease. Semin Pediatr. Surg. 19 (3), 194–200. 10.1053/j.sempedsurg.2010.03.004 20610192

[B17] Kerem E. Hirawat S. Armoni S. Yaakov Y. Shoseyov D. Cohen M. (2008). Effectiveness of PTC124 treatment of cystic fibrosis caused by nonsense mutations: a prospective phase II trial. Lancet (London, England) 372 (9640), 719–727. 10.1016/S0140-6736(08)61168-X 18722008

[B18] Kjaer S. Ibanez C. F. (2003). Intrinsic susceptibility to misfolding of a hot-spot for Hirschsprung disease mutations in the ectodomain of RET. Hum. Mol. Genet. 12 (17), 2133–2144. 10.1093/hmg/ddg227 12915470

[B19] Leon T. Y. So M. T. Lui V. C. Hofstra R. M. Tam P. K. Ngan E. S. (2012). Functional analyses of RET mutations in Chinese Hirschsprung disease patients. Birth Defects Res. Part A: Clin. Mol. Teratol. 94 (1), 47–51. 10.1002/bdra.22863 22131258

[B20] McWilliam S. J. Antoine D. J. Smyth R. L. Pirmohamed M. (2017). Aminoglycoside-induced nephrotoxicity in children. Pediatr. Nephrol. 32 (11), 2015–2025. 10.1007/s00467-016-3533-z 27848094 PMC5624973

[B21] Muniz M. Riezman H. (2016). Trafficking of glycosylphosphatidylinositol anchored proteins from the endoplasmic reticulum to the cell surface. J. Lipid Res. 57 (3), 352–360. 10.1194/jlr.R062760 26450970 PMC4767001

[B22] Plaza-Menacho I. Burzynski G. M. de Groot J. W. Eggen B. J. Hofstra R. M. (2006). Current concepts in RET-related genetics, signaling and therapeutics. Trends Genet. 22 (11), 627–636. 10.1016/j.tig.2006.09.005 16979782

[B23] Pranke I. Bidou L. Martin N. Blanchet S. Hatton A. Karri S. (2018). Factors influencing readthrough therapy for frequent cystic fibrosis premature termination codons. ERJ Open Res. 4 (1). 10.1183/23120541.00080-2017 PMC5827411 29497617

[B24] Prokhorova I. Altman R. B. Djumagulov M. Shrestha J. P. Urzhumtsev A. Ferguson A. (2017). Aminoglycoside interactions and impacts on the eukaryotic ribosome. Proc. Natl. Acad. Sci. U. S. A. 114 (51), E10899–E1E908. 10.1073/pnas.1715501114 29208708 PMC5754804

[B25] Schriemer D. Sribudiani Y. Ijpma A. Natarajan D. MacKenzie K. C. Metzger M. (2016). Regulators of gene expression in enteric neural crest cells are putative Hirschsprung disease genes. Dev. Biol. 416 (1), 255–265. 10.1016/j.ydbio.2016.06.004 27266404

[B26] Sergi C. (2015). Hirschsprung's disease: Historical notes and pathological diagnosis on the occasion of the 100(th) anniversary of Dr. Harald Hirschsprung's death. World J. Clin. Pediatr. 4 (4), 120–125. 10.5409/wjcp.v4.i4.120 26566484 PMC4637802

[B27] Shimizu-Motohashi Y. Komaki H. Motohashi N. Takeda S. Yokota T. Aoki Y. (2019). Restoring dystrophin expression in Duchenne muscular dystrophy: current status of therapeutic approaches. J. Personal. Med. 9 (1). 10.3390/jpm9010001 PMC6462907 30621068

[B28] Sribudiani Y. Chauhan R. K. Alves M. M. Petrova L. Brosens E. Harrison C. (2018). Identification of variants in RET and IHH pathway members in a large family with history of Hirschsprung disease. Gastroenterology 155 (1), 118–29 e6. 10.1053/j.gastro.2018.03.034 29601828

[B29] Traugott A. L. Moley J. F. (2010). The RET protooncogene. Cancer Treat. Res. 153, 303–319. 10.1007/978-1-4419-0857-5_17 19957232

[B30] Widowati T. Melhem S. Patria S. Y. de Graaf B. M. Sinke R. J. Viel M. (2016). RET and EDNRB mutation screening in patients with Hirschsprung disease: functional studies and its implications for genetic counseling. Eur. J. Hum. Genet. 24 (6), 823–829. 10.1038/ejhg.2015.214 26395553 PMC4867453

[B31] Yu H. Liu X. Huang J. Zhang Y. Hu R. Pu J. (2014). Comparison of read-through effects of aminoglycosides and PTC124 on rescuing nonsense mutations of HERG gene associated with long QT syndrome. Int. J. Mol. Med. 33 (3), 729–735. 10.3892/ijmm.2013.1601 24366185

