# Supplementary material for: Functional Studies on Novel RET Mutations and Their Implications for Genetic Counseling for Hirschsprung Disease
Source: Front Genet. 2019 Oct 8;10:924. doi: 10.3389/fgene.2019.00924 (PMC6792140; doi:10.3389/fgene.2019.00924)
Supplement: Supplementary file 1 [file Table_1.docx]

Supplementary Material

# Supplementary Tables

**Table S1. Primers used in this study**

| **Primer** | **Sequence (5' to 3')** | **Purification method** |
| --- | --- | --- |
| c229t_sense | tgcagccgtgtgcagtacgtgccgtag | ULTRAPAGE |
| c229t_antisense | ctacggcacgtactgcacacggctgca | ULTRAPAGE |
| g254a_sense | cctcctggatgcagatctagttgttctcatgcagc | ULTRAPAGE |
| g254a_antisense | gctgcatgagaacaactagatctgcatccaggagg | ULTRAPAGE |
| g754t_antisense | ccatcaccacctactcgcgcgcgcc | ULTRAPAGE |
| g754t_sense | ggcgcgcgcgagtaggtggtgatgg | ULTRAPAGE |
| c789g_antisense | gtcctcgtcctacacggtcaccggga | ULTRAPAGE |
| c789g_sense | tcccggtgaccgtgtaggacgaggac | ULTRAPAGE |
| c2308t_antisense | gacagcaggtctcaaagctcactcgggg | ULTRAPAGE |
| c2308t_sense | ccccgagtgagctttgagacctgctgtc | ULTRAPAGE |
| c2578t_antisense | ctgcatcccctatgagatctgccaggcaaatga | ULTRAPAGE |
| c2578t_sense | tcatttgcctggcagatctcataggggatgcag | ULTRAPAGE |
| del2333-antisense | gttgacctgcttcaggcgttgaactctgacag | ULTRAPAGE |
| del2333_sense | ctgtcagagttcaacgcctgaagcaggtcaac | ULTRAPAGE |
| ins_3nt_after_196-antisense | ccaggcggaggaagctgggcacctcctca | ULTRAPAGE |
| ins_3nt_after_196_sense | tgaggaggtgcccagcttcctccgcctgg | ULTRAPAGE |
| RET_c166a | tacgtccatgccatgcgggacgccc | ULTRAPAGE |
| RET_c166a_as | gggcgtcccgcatggcatggacgta | ULTRAPAGE |
| RET_a3185t as | gggaaattctaccaaagagtttgttttcaatccatgtggaag | ULTRAPAGE |
| RET_a3185t_s | cttccacatggattgaaaacaaactctttggtagaatttccc | ULTRAPAGE |
| RET c2802-2AtoG F1 | ggcaattgaatccctttttg | ULTRAHAP |
| RET c2802-2AtoG R1 | cttccagcattgcagcatc | ULTRAHAP |

**Table S2. Antibodies used in this study**

| **Antibody** | | **Source** | **Identifier** | **Host** | **Dilution** |
| --- | --- | --- | --- | --- | --- |
| anti-RET | Santa Cruz | | sc-365943 | Mouse | 1:500 for WB;  1:100 for IF |
| anti-P-RET | Abcam | | ab51103 | Rabbit | 1:1000 |
| anti-MYC | Abcam | | ab32 | Mouse | 1:1000 |
| anti-STAT3 | Santa Cruz | | sc-8019 | Mouse | 1:500 |
| anti-P-STAT3 | Santa Cruz | | sc-8059 | Mouse | 1:500 |
| anti-ERK | Cell Signaling | | 4695 | Rabbit | 1:1000 |
| anti-P-ERK | Cell Signaling | | 9101 | Rabbit | 1:1000 |
| anti-GAPDH | Proteintech | | 60004-1-Ig | Mouse | 1:5000 |
| anti-GDNF | Santa Cruz | | sc-398555 | Mouse | 1:200 |
| anti-GFP | OriGene Technologies, | | [TA150052](http://www.origene.com.cn/antibody/antitag_detail.aspx?sku=TA150052) | Mouse | 1:2000 |
| Rhodamine Phalloidin | Cytoskeleton | | PHDR1 | - | 100 nm |
| Alexa Fluor® 488 - Conjugated Goat anti-Mouse IgG (H+L) | ZSGB-BIO Beijing China | | ZF-0512 | - | 1:200 |
| Peroxidase-Conjugated Goat anti-Mouse IgG (H+L) | ZSGB-BIO Beijing China | | ZB-2301 | - | 1:5000 |
| Peroxidase-Conjugated Goat anti-Mouse IgG (H+L) | ZSGB-BIO Beijing China | | ZB-2305 | - | 1:5000 |

**Table S3. Agents used in this study**

| **Agents** | **Source** | **Identifier** |
| --- | --- | --- |
| Cycloheximide | MedChem Express | HY-12320 |
| G418 | MedChem Express | HY-17561 |
| PTC124 | MedChem Express | HY-14832 |
